# Follow-Up Study of Subdermal Low-Echoic Lesions in the Ischial Region in Wheelchair Users With Spinal Cord Injuries

**DOI:** 10.3389/fmed.2022.848338

**Published:** 2022-03-09

**Authors:** Shinji Kawasaki, Yukihide Nishimura, Ken Kouda, Yasunori Umemoto, Tokio Kinoshita, Takamasa Hashizaki, Makoto Kawanishi, Taro Nakamura, Fumihiro Tajima

**Affiliations:** ^1^Department of Rehabilitation Medicine, Wakayama Medical University, Wakayama, Japan; ^2^Division of Rehabilitation, Wakayama Medical University Hospital, Wakayama, Japan; ^3^Department of Rehabilitation Medicine, Iwate Medical University, Iwate, Japan; ^4^Department of Rehabilitation Medicine, Oita Nakamura Hospital, Oita, Japan

**Keywords:** pressure injury, spinal cord, ultrasonography, sitting time, interface pressure

## Abstract

**Objective:**

To follow up patients with spinal cord injuries with subdermal low-echoic lesions in the ischial region for abnormalities after 1 year.

**Design:**

A retrospective cohort study.

**Setting:**

A Japanese rehabilitation center.

**Participants:**

We included patients with chronic spinal cord injuries and subdermal low-echoic lesions who underwent routine inspection and palpation examinations (*n* = 7).

**Interventions:**

Education on pressure injury and instruction on pressure relief and seating was provided and the patients were followed up for abnormalities after 1 year. Self-reports were obtained on wheelchair sitting time, and interface pressure was recorded while the patients were seated on the wheelchair. Interface pressure measurements at the bilateral ischial regions were recorded with a force-sensitive application pressure mapping system.

**Outcome Measures:**

The primary outcome was the presence of subdermal low-echoic lesions in the bilateral ischial regions on ultrasonography at the 1-year follow-up examination. Secondary outcomes included wheelchair sitting time and interface pressure in the bilateral ischial regions.

**Results:**

Of the 10 areas that showed subdermal low-echoic lesions on ultrasonography, nine had improved after 1 year. One area that did not improve was an open wound. At the follow-up examination, the pressure duration was reduced in all patients, and the interface pressure could be reduced in 5/7 patients.

**Conclusions:**

This is the first study to follow up with patients having spinal cord injuries and subdermal low-echoic lesions in the ischial region using ultrasonography. The low-echoic lesions improved within 1 year by reducing the pressure duration and interface pressure. Pressure injury prevention in patients with spinal cord injuries relies on the early detection of skin abnormalities, and education and instruction to change self-management behaviors are recommended.

## Introduction

A pressure injury (PI) is defined as localized damage to the skin and underlying soft tissue, usually over a bony prominence, and occurs due to intense or prolonged pressure or pressure in combination with shear forces (1). There is an inverse relationship between pressure and time to tissue damage, and different types of tissues have different susceptibilities to ischemia (2–4). Deeper tissues, such as the muscle, are reportedly more susceptible to pressure-related ischemia than the skin (5, 6). DeLisa and Mikulic (7) reported that “the visible ulcer represents only the tip of the iceberg or the apex of the lesion” and pointed out that underlying soft tissue damage may occur even when the skin is visually intact.

The bottom-up theory that most pressure-induced tissue damage results from underlying soft tissue damage is supported by a considerable body of clinical and experimental evidence (8–10). The importance of early diagnosis of underlying soft tissue injury is being recognized, and ultrasonography is being used in clinical practice to evaluate the underlying soft tissues (10, 11). Kanno et al. (11) have reported the benefits of early detection of skin abnormalities underneath by ultrasonography in patients with chronic spinal cord injuries (SCIs) by performing PI examination using combined inspection, palpation, and ultrasonography.

The ischial region is the main weight-bearing region during sitting and must withstand mechanical stress (12). In active SCIs, a PI most frequently appears in the skin over the ischial region (13, 14). We reported that in chronic SCI patients who use wheelchairs daily, the areas with subdermal low-echoic lesions identified by ultrasonography were those with long pressure durations and high interface pressures (15).

We conducted PI examinations on a regular basis, and physicians and therapists provided education and instructions on pressure relief and sheeting. However, it is unclear whether the early detection of skin abnormalities by ultrasonography and early intervention would prevent the transition to PI. Therefore, this study aimed to follow up with patients having chronic SCIs with subdermal low-echoic lesions in the ischial region for abnormalities for 1 year.

## Materials and Methods

### Participants

**Figure 1:**
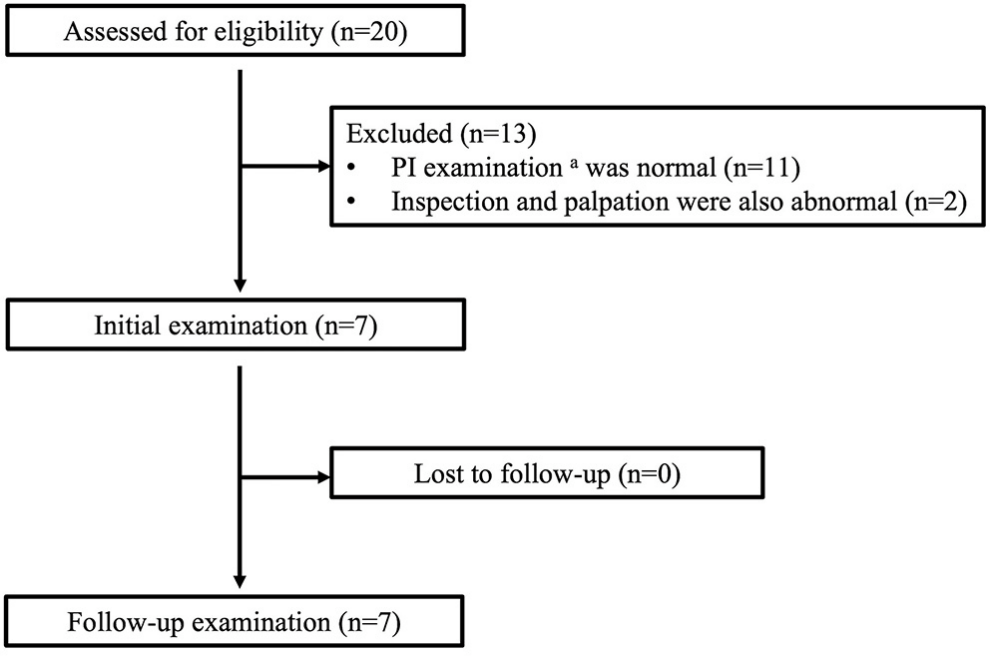
The flow of participants through the study. ^a^PI examination consists of inspection, palpation, and ultrasonography of the bilateral ischial regions.

The study participants were 20 patients with chronic traumatic SCI who participated in the PI examination held before the Oita International Wheelchair Marathon from 2010 to 2014. Of these, seven patients who underwent routine inspection and palpation and in whom subdermal abnormalities were found by ultrasonography were retrospectively selected (Figure 1). The exclusion criteria were age <18 years, cause of injury other than trauma, a minimum of 2 years since injury, a primary mode of transportation other than a wheelchair, and a C-shaped kyphotic posture (16, 17).

Table 1 shows the patient's sex, age, height, weight, body mass index (BMI), time since injury, and level of neurological injury. Two physicians independently assessed the extent of neurological injury in each case, and all patients were completely paralyzed. All patients could perform push-ups for pressure relief and manage their own transfer from their wheelchair.

**Table 1:** Participants' characteristics.

**Case**	**Sex**	**Age (years)**	**Height (cm)**	**Body mass (kg)**	**BMI (kg/m^**2**^)**	**Time since injury (years)**	**Neurological level**	**AIS**
1	M	60	160	53	20.7	26	C8	A
2	M	51	171	65	22.0	30	Th8	A
3	M	43	182	83	25.1	16	C7	A
4	M	61	164	47	17.5	26	Th9	A
5	M	60	162	65	24.8	46	Th11	A
6	M	55	171	53	17.9	27	Th6	A
7	M	48	168	60	21.3	20	Th7	A

*AIS, ASIA impairment scale; ASIA, American Spinal Cord Injury Association; BMI, body mass index*.

The study protocol was approved by the Human Ethics Review Committee of Wakayama Medical University (Wakayama, Japan), and informed consent was obtained from all participants.

### Outcome Measures

The primary outcome was the presence of subdermal low-echoic lesions in the bilateral ischial regions on ultrasonography at the 1 year follow-up examination. The secondary outcomes were wheelchair sitting time and interface pressure during the resting position during the initial and follow-up examinations.

### Protocol

**Figure 2:**
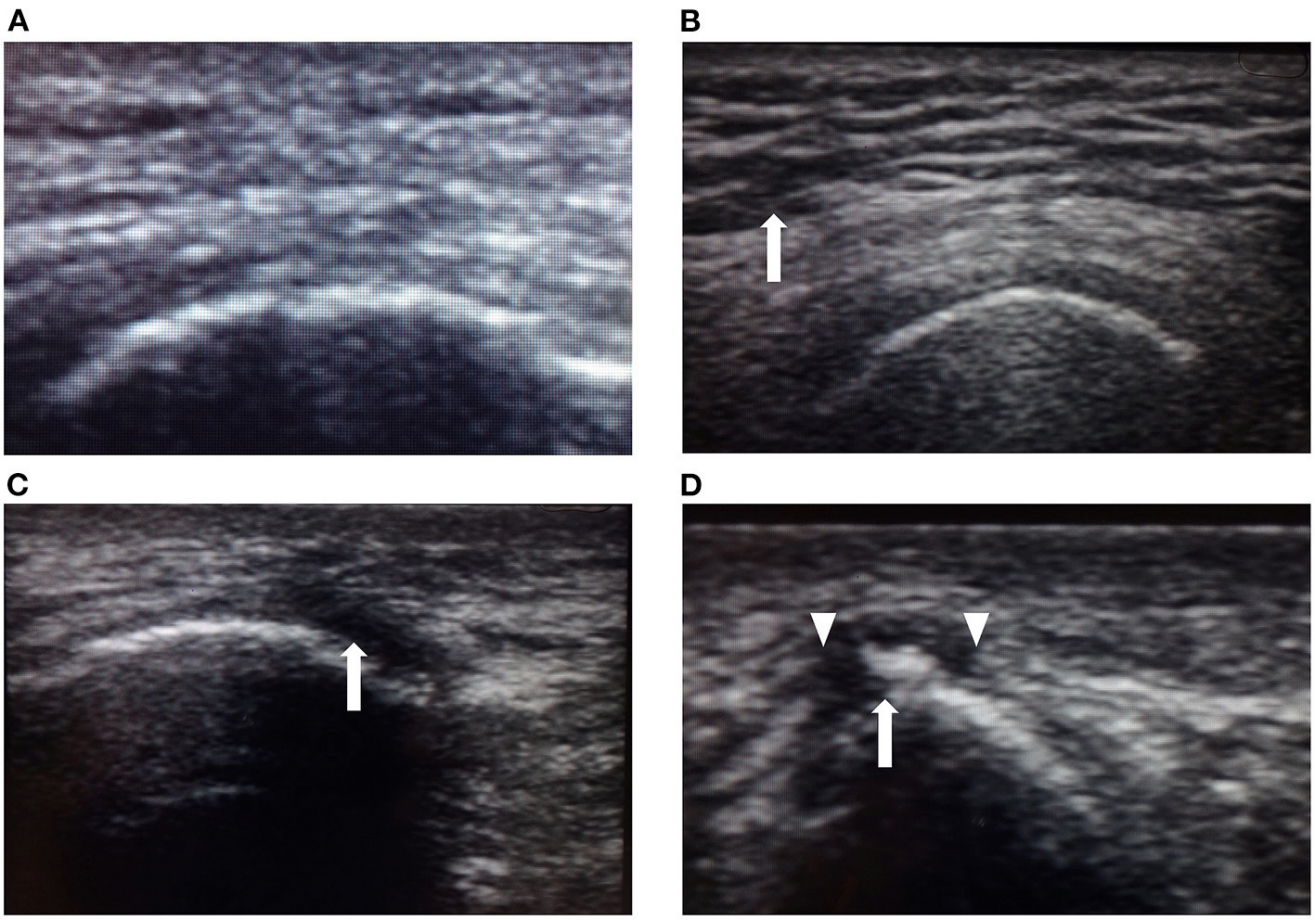
Typical ultrasound images. **(A)** Ultrasound image of normal skin showing a homogeneous pattern of ultrasonic reflections from the epidermis to the right ischial bone (arrow), with a clear layer of muscle. **(B)** Unclear layered structure with a coarse echo texture (arrow) is most frequently seen even in areas where hypoechoic lesions are not present and judged separately from low-echoic lesions. **(C)** Low-echoic lesion near the right ischial region in Case 2. The image shows a hypoechoic area near the bone (arrow). This hypoechoic area did not disappear when compressed. **(D)** Low-echoic lesion near the right ischial region in Case 7. Heterogeneous hypoechoic areas contained high-echoic areas (arrow). The image shows an unclear layered structure and discontinuous fascia. Discontinuous fascia shows a broken or discontinuous line with heterogeneous echo signals (triangle).

The PI examination used the testing protocol previously described by Kanno et al. (11). Each patient was examined in the prone position as follows: First, one examiner carefully inspected the skin of the right and left ischial regions for the presence of redness, areas of localized purple or chestnut coloring, swelling, or epidermal freshness, excluding old scars or pigmentation due to old wounds. Second, the examiner palpated the same areas to assess localized heat or floating sensation. Third, the same areas were imaged using a high-frequency linear ultrasound array. A single examiner scanned three different aspects to detect both hypo- and hyperechoic lesions in B-mode images from the skin to the bone. The areas examined were classified as normal or abnormal by ultrasound by another physician who was not informed of the diagnosis based on inspection and palpation. Typical ultrasound images of the lesions are shown in Figure 2. Normal lesion areas showed a homogeneous pattern of ultrasound reflections from the epidermis to the bone, with a clearly defined muscle layer. Contrastingly, low-echoic lesions were heterogeneous, with either little or no echogenic signal, which we defined as hypoechoic lesions. Aoi et al. (8) reported four abnormal signs found in deep tissue injury: unclear layered structure, hypoechoic lesion, discontinuous fascia, and heterogeneous hypoechoic area. In our examination, unclear layered structures were often found even in areas where hypoechoic lesions were not present, and judged separately from low-echoic lesions.

Patients were asked a series of standardized questions to collect data on the descriptive characteristics of their activities of daily living. The physician and therapist interviewed the patients directly to obtain information on wheelchair sitting time per day, uninterrupted sitting time, and pressure duration. Uninterrupted sitting time was defined as the longest time spent without getting out of the wheelchair. The pressure duration was defined as the longest time spent in a wheelchair without pressure relief. We listened to the conscious pressure relief interval with a push-up.

To measure the interface pressure at bilateral ischial regions, the patients sat in their own wheelchair equipped with a cushion, and the seat mat was placed on the cushion. The patients sat looking straight ahead with their arms on their thighs. Pressure data were recorded for 3 s at a sampling rate of 10 Hz. The apexes of the left and right ischial regions were palpated to identify where the most pressure was applied to the seat mat. The interface pressure was defined as the value recorded by one sensor (coinciding with a 2.38 × 2.38 cm area) at the highest pressure within the ischial regions (18).

### Instrumentation

Ultrasound images were collected using a portable ultrasound system (MicroMaxx, SonoSite, Bothell, WA, USA) equipped with a 10 MHz probe. This has been found to provide reasonable resolution for images 20–30 mm below the skin's surface (19). Interface pressure measurements at the bilateral ischial regions were recorded with a force-sensitive application pressure mapping system (Vista Medical, Winnipeg, Manitoba, Canada). The seat mat (consisting of a 16 × 16 array of pressure sensors, size 2.38 × 2.38 cm) was calibrated according to the manufacturer's instructions prior to each measurement day and calibrated using the plane load method (range, 0–200 mmHg).

### Prevention Education and Instruction

After the PI examination, the physician provided basic education to the patients regarding PI development. The therapist also provided pressure relief instructions and seating with the visual feedback of the interface pressure image. Specifically, the therapists recommended pressure relief every 15–30 min, as recommended by the Pressure Ulcer Prevention Guidelines (20), and advised the patients to use a timer to perform push-up pressure relief. In addition, other pressure relief methods included individual evaluation and instruction, such as trunk forward and lateral tilting, while reviewing the pressure interface image. Seating changes included changing the type of wheelchair cushion and adjusting the air volume and fluid to provide an optimal seating support surface.

## Results

### Ultrasound for PI Examination

Ten of the 14 examined areas showed subdermal low-echoic lesions on ultrasonography at the initial examination. Additionally, 9/10 areas showed abnormal findings only on ultrasonography, while inspection and palpation results were normal. The remaining area was an open wound that was also noted to be abnormal on inspection and palpation (Table 2). Unclear layered structures were found in all 14 areas. The low-echoic lesions were located deeply adjacent to the bone and showed discontinuous fascia. Of the 10 low-echoic lesions, 3 (30%) were heterogeneous hypoechoic areas also containing hyperechoic areas.

**Table 2:** Initial pressure injury examination of the study participants.

**Case**	**Rt ischial region**			**Lt ischial region**		
	**Inspection**	**Palpation**	**Ultrasound**	**Inspection**	**Palpation**	**Ultrasound**
1	Normal	Normal	Low-echoic lesion	Normal	Normal	Low-echoic lesion
2	Normal	Normal	Low-echoic lesion	Normal	Normal	Low-echoic lesion
3	Normal	Normal	Low-echoic lesion	Normal	Normal	Normal
4	Normal	Normal	Low-echoic lesion	Normal	Normal	Normal
5	Normal	Normal	Normal	Normal	Normal	Low-echoic lesion
6	Normal	Normal	Normal	Normal	Normal	Low-echoic lesion
7	Normal	Normal	Low-echoic lesion	Wound^a^	Free-floating^b^	Low-echoic lesion

*Rt, right; Lt, left*.

a *This area was an open wound with redness and ulceration on inspection*.

b *This area had a free-floating sensation around the open wound on palpation*.

The results of the ultrasound examinations at 1 year follow-up are shown in Table 3. Of the 10 areas that showed low-subdermal echoic lesions underneath the skin on ultrasonography, nine had improved after 1 year. The one without improvement was an open wound that showed abnormalities on inspection and palpation.

**Table 3:** Ultrasound for the examination of pressure injury.

**Case**	**Rt ischial region**		**Lt ischial region**	
	**Initial**	**Follow-up**	**Initial**	**Follow-up**
1	Low-echoic lesion	Normal	Low-echoic lesion	Normal
2	Low-echoic lesion	Normal	Low-echoic lesion	Normal
3	Low-echoic lesion	Normal	Normal	Normal
4	Low-echoic lesion	Normal	Normal	Normal
5	Normal	Normal	Low-echoic lesion	Normal
6	Normal	Normal	Low-echoic lesion	Normal
7	Low-echoic lesion	Normal	Open wound^a^	Open wound^a^

*Rt, right; Lt, left*.

a *This area was open wound with redness and ulceration on inspection, and had a free-floating sensation around the open wound on palpation*.

### Wheelchair Sitting Time

The results of wheelchair sitting time per day, uninterrupted sitting time, and pressure duration are shown in Table 4. Wheelchair sitting time per day did not change between the initial and follow-up examinations in all cases. Uninterrupted sitting time was reduced in two patients (Cases 1 and 7). These two patients could get out of their wheelchairs once or twice a day and spend time in bed. The pressure duration was reduced in all cases, and 3/7 patients did not push up for more than 60 min while in the wheelchair.

**Table 4:** Wheelchair sitting time.

**Case**	**Sitting time per day (h)**		**Uninterrupted sitting time (h)**		**Pressure duration (min)**	
	**Initial**	**Follow-up**	**Initial**	**Follow-up**	**Initial**	**Follow-up**
1	10	10	10	4	120	60
2	15	15	5	5	60	30
3	15	15	8	8	180	30
4	16	16	4	4	240	120
5	16	16	7	7	240	120
6	12	12	4	4	240	30
7	18	18	7	4	60	30

### Pressure Mapping Systems

**Figure 3:**
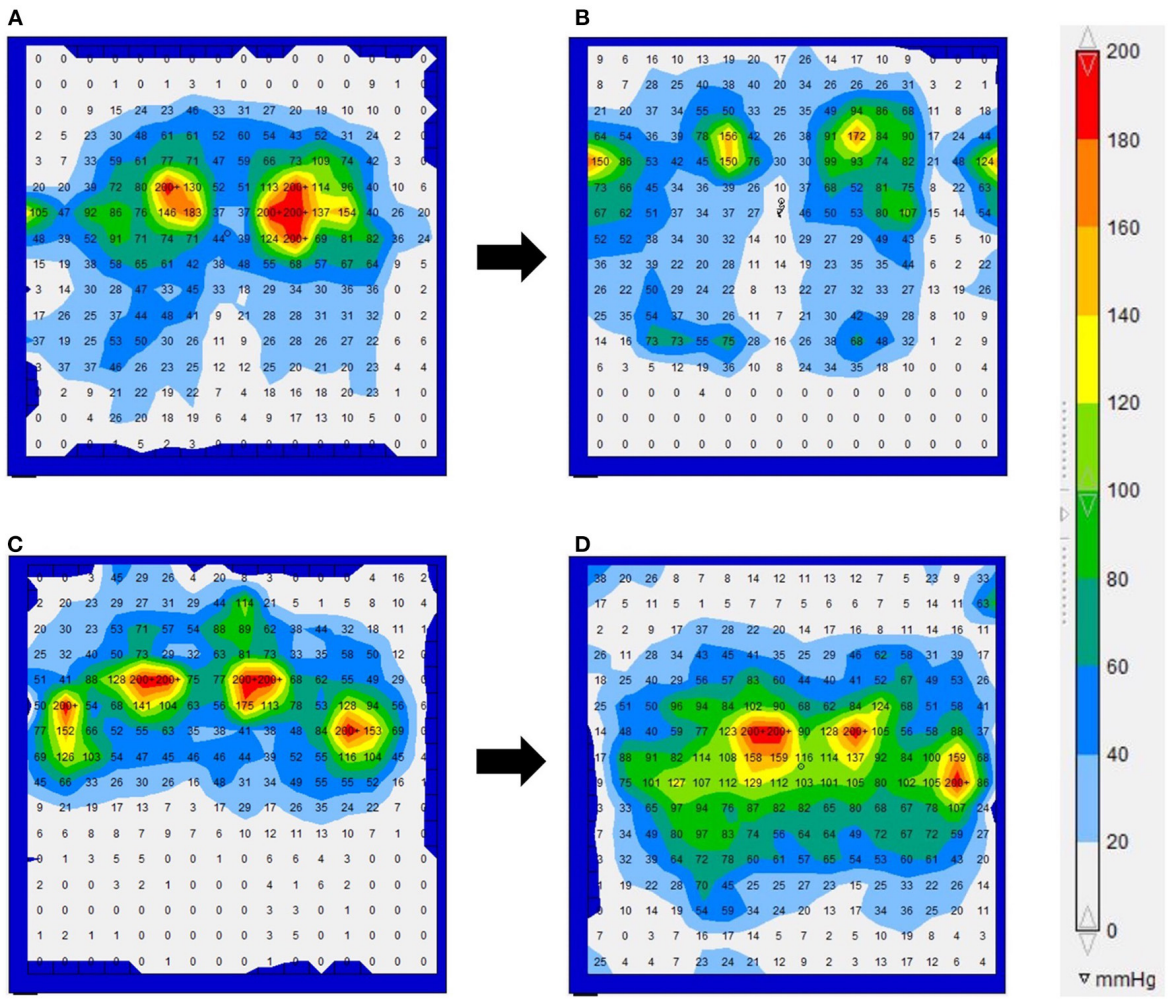
The interface pressure for the force-sensitive applications pressure mapping system in Cases 2 **(A,B)** and 7 **(C,D)**. **(A)** Upon examination, the interface pressure near the bilateral ischial regions exceeded the maximum measured value of 200 mmHg. **(B)** A 1 year follow-up examination. The interface pressure decreased by changing from the form cushion to the ROHO cushion. **(C)** At the initial examination, the interface pressure near the bilateral ischial regions and greater trochanter exceeded the maximum measured value of 200 mmHg. **(D)** A 1 year follow-up examination. Even if the cushion type was changed or adjusted, the interface pressure in the ischial region remained above the maximum value.

The results of the interface pressures in the bilateral ischial regions and the cushions used during the measurements are shown in Table 5. The interface pressures at the ischial region areas that showed low-echoic lesions underneath the skin on ultrasonography were above 200 mmHg. In 5/7 patients, the interface pressure could be reduced at the follow-up examination. The interface pressure was not reduced during the intervention in the remaining two patients and remained above 200 mmHg at the follow-up examination. Figure 3 shows the results of Case 2 (Figures 3A,B), in which the interface pressure could be reduced, and Case 7 (Figures 3C,D), in which the pressure could not be reduced.

**Table 5:** Interface pressure and cushion type.

**Case**	**Rt interface pressure (mmHg)**		**Lt interface pressure (mmHg)**		**Type of cushion**	
	**Initial**	**Follow-up**	**Initial**	**Follow-up**	**Initial**	**Follow-up**
1	>200^a^	137	>200^a^	160	Other air cushion	ROHO cushion^c^
2	>200^a^	156	>200^a^	172	Form cushion	ROHO cushion^c^
3	>200^a^	105	128	115	ROHO cushion	ROHO cushion^d^
4	>200^a^	137	>200	135	Form cushion	ROHO cushion^c^
5	>200	131	>200^a^	127	Jay 2 cushion	Jay 2 cushion^d^
6	>200	>200	>200^a^	>200	ROHO cushion	ROHO cushion^e^
7	>200^a^	>200	>200^b^	>200	ROHO cushion	ROHO cushion^e^

*Rt, right; Lt, left*.

a *These areas were classified as low-echoic lesions and showed no lesions on inspection or palpation*.

b *This area shows wound, free-floating sensation, and low-echoic lesions*.

c *Changed to ROHO cushion (Quadtro Select mid profile)*.

d *Instructed how to adjust cushion air and fluid placement*.

e *Changed to ROHO cushion (Quadtro Select high profile) and instructed how to adjust the cushion air*.

## Discussion

This study demonstrated that subdermal low-echoic lesions detected by ultrasonography could be improved after 1 year by reducing the pressure duration and interface pressure at the ischial region. In the clinical management of patients with SCI, the early detection of skin abnormalities and early reduction of pressure duration and interface pressure are important for the prevention and improvement of PIs.

The most important early intervention is immediate pressure relief in the ischial region, and the Pressure Ulcer Prevention Guidelines recommend pressure relief every 15–30 min (20). In an animal model developed by Gabriel et al. (21) it was reported that complete venous occlusion for as short a period of 10 min was detrimental to tissues and irreversible damage could occur within 60 min in a skeletal muscle model. During this study's initial examinations, no patient performed push-up pressure relief at the recommended time. Additionally, two patients did not perform any conscious pressure relief during the 4 h they remained in their wheelchair. The PI examination and educational instruction interventions reduced the pressure duration in all patients, although the recommended time was not met in some at the follow-up examination. At the stage of low-echoic lesions underneath the skin, conscious push-up relief at least once every 30 min may prevent progression to PIs.

Additionally, the provision and evaluation of optimal seating support surfaces, such as wheelchair cushions, is a vital intervention method. Interface pressure measurement with a pressure distribution device is the most commonly used parameter to assess pressure dispersion in the buttocks (22). Muscle atrophy in the paralyzed region of the SCI decreased the ground contact area and increased the absolute pressure per load area. It has been reported that the interface pressure is higher in people with SCIs than in healthy patients (23, 24). This study also showed that the interface pressure at the initial examination was more than 200 mmHg in all patients. Although there is still a lack of consensus on the critical pressure value at which PI occurs, the general recommendation is that tissues be exposed to the lowest possible pressure (25, 26). In this study, interface pressure could be reduced in three patients who changed the type of cushion and in two patients who were instructed on how to adjust the cushion.

On the other hand, in the remaining two patients, even after changing to the ROHO cushion (Quadtro Select high profile) (26) suitable for interface pressure dispersion, the interface pressure remained above 200 mmHg. These patients were instructed to use a timer to tightly control the frequency of pressure relief. Thorough pressure relief was performed for those patients who could not easily reduce their own interface pressure, which was confirmed to improve.

Subcutaneous tissue injuries are often difficult to diagnose by inspection and palpation. Magnetic resonance imaging (MRI) is a currently recommended imaging modality for measuring anatomical features associated with subcutaneous tissue injuries (27–31). However, MRI has three major clinical drawbacks: cost, time, and convenience, and it is not a viable assessment tool in routine clinical practice. Ultrasonography may be a viable alternative to overcome the disadvantages of MRI (8, 11, 31–34), as ultrasound devices are portable, widely used in clinical situations, non-invasive, and can detect subcutaneous conditions in real-time. In this study, as only subdermal abnormalities were detected, low-echoic lesions in all patients improved within 1 year by reducing the pressure applied to the tissue and the amount of time this was applied.

On the other hand, the open wound area took more than 2 years to heal. Ultrasound allows for the early and cost-effective detection of skin abnormalities, facilitating early intervention and potentially preventing progression to PI. Visualizing abnormal tissue may also motivate patients to change their self-management behaviors. We recommend regular ultrasonography examination in daily clinical practice to detect subcutaneous tissue damage in patients with chronic SCIs.

The primary limitation of this study was the small sample size. A prospective study using a larger sample size is recommended to clarify whether ultrasound underneath the skin abnormalities is useful in the early detection and prevention of PI. Additionally, the 10 MHz probes appear to have low sensitivity for differentiating dermal and hypodermal alterations. Perhaps, this data can be validated with a future study using higher frequency probes—ideal for studying the dermis. The force-sensitive application pressure sensor could not distinguish pressures >200 mmHg. Therefore, the ischial interface pressure at the initial and follow-up examinations may have been underestimated. Finally, the fact that pressure duration was subjectively assessed and based on a self-report questionnaire is another limitation of this study. There is a need for the sustained recording of the interface pressure and pressure relief intervals to provide the true pressure duration.

## Conclusions

To our knowledge, this is the first study to follow the course of subdermal low-echoic lesions in the ischial region by ultrasonography in patients with SCI. In our study, as only subdermal abnormalities were detected, low-echoic lesions in all patients improved within 1 year by reducing the pressure applied to the tissue and the amount of time this was applied. Based on these findings, the prevention of PI in patients with SCI is recommended to include early detection of skin abnormalities and education and instruction regarding self-management behaviors.

## Data Availability Statement

The raw data supporting the conclusions of this article will be made available by the authors, without undue reservation.

## Ethics Statement

The study protocol was approved by the Human Ethics Review Committee of Wakayama Medical University (Wakayama, Japan). The patients/participants provided their written informed consent to participate in this study.

## Author Contributions

SK and YN conceptualized and designed the study, drafted the initial manuscript, and reviewed and revised the manuscript. KK, YU, TK, TH, MK, and TN designed the data collection instruments, collected data, carried out the initial analyses, and reviewed and revised the manuscript. FT designed the data collection instruments, coordinated and supervised data collection, and critically reviewed the manuscript. All authors approved the final manuscript as submitted and agree to be accountable for all aspects of the work.

## Conflict of Interest

The authors declare that the research was conducted in the absence of any commercial or financial relationships that could be construed as a potential conflict of interest.

## Publisher's Note

All claims expressed in this article are solely those of the authors and do not necessarily represent those of their affiliated organizations, or those of the publisher, the editors and the reviewers. Any product that may be evaluated in this article, or claim that may be made by its manufacturer, is not guaranteed or endorsed by the publisher.
